# Establishing A Memory Clinic Biobank Study

**DOI:** 10.1002/alz70856_106651

**Published:** 2026-01-08

**Authors:** Bernadette McGuinness, Claire Lewis, Gareth McKeeman, Katherine Patterson, Kathryn Ryan, Emma L Cunningham

**Affiliations:** ^1^ Memory Clinic, Belfast Health and Social Care Trust, Belfast, United Kingdom; ^2^ Queen's University Belfast, Belfast, Northern Ireland, United Kingdom; ^3^ Northern Ireland Biobank, Belfast, United Kingdom; ^4^ Belfast Trust, Belfast, United Kingdom; ^5^ Queen's University Belfast, Belfast, United Kingdom

## Abstract

**Background:**

The Northern Ireland Biobank was established to facilitate research access to quality‐assured biological samples. Patients, with capacity to consent, are invited to give written consent for collection and retention of clinically surplus material for the purposes of research. In addition, blood can be collected for the purposes of biobanking.

**Method:**

Since May 2022 patients have been invited to donate clinically surplus cerebrospinal fluid (CSF) to the NI Biobank. Since March 2023 patients have been invited to donate blood for the purposes of biobanking. CSF is collected using the drip method into false‐bottomed Starstedt tubes. Blood is collected, non‐fasting, into two EDTA bottles. Both are transported at room temperature to Trust (CSF) and NI Biobank (blood) laboratories on the same day. CSF is frozen at ‐80C. Blood is processed within 24 hours and stored as plasma and buffy coat.

**Result:**

Thus far, *n* = 37 participants have consented to the retention of their clinical surplus CSF. In addition, *n* = 105 participants have donated blood for the purposes of biobanking.

The main barrier to recruitment has been the availability of staff time to facilitate the consent and sampling processes.

**Conclusion:**

Biobanking of biological samples, with access to associated clinical data, provides opportunities to study real‐world memory clinic cohorts. Researchers interested in accessing these samples should contact the authors.

